# A convenient catalyst system for microwave accelerated cross-coupling of a range of aryl boronic acids with aryl chlorides

**DOI:** 10.1186/1860-5397-3-18

**Published:** 2007-05-30

**Authors:** Matthew L Clarke, Marcia B France, Jose A Fuentes, Edward J Milton, Geoffrey J Roff

**Affiliations:** 1 School of Chemistry, University of St Andrews, EaStCHEM, St Andrews, Fife, UK; 2 Department of Chemistry, Washington and Lee University, Lexington, VA 24450, USA

## Abstract

A convenient microwave accelerated cross-coupling procedure between aryl chlorides with a range of boronic acids has been developed. An explanation for the low reactivity of highly fluorinated boronic acids in Suzuki coupling is provided.

## Background

The Suzuki cross-coupling represents an extremely useful method for biaryl synthesis and is widely applied in organic chemistry. [1–6] In the last 10 years, there has been intense research interest in Suzuki reactions of aryl chloride substrates since these substrates are cheaper and more widely available than aryl bromides. A range of catalysts now exist for aryl chloride activation, including diphosphine-Pd catalysts,[7] mono-phosphine Pd catalysts, [8–9] cyclometallated Pd precursors,[10] and potentially hemi-labile bidentate ligands. [11–15] Some years ago, we demonstrated that the palladium complexes formed from the amine-phosphine ligand, **dcpmp**, **1** gave very active catalysts for this reaction[13]. At the time this system was one of the few catalysts capable of cross-coupling unactivated aryl chlorides below 100°C. Reaction times were typically 12 hours or slightly less.

More recently, advances in chemical microwave technology have stimulated considerable research on microwave accelerated cross-coupling reactions. [16–27] The reduction of reaction times to 20 minutes or less has made the reaction considerably more valuable in diversity orientated synthesis and med-chem optimisation studies. A small number of these catalyst systems have been found to deliver microwave accelerated aryl chloride cross-coupling, and in the course of another project, we discovered that the Pd/dcpmp catalyst system was just such a catalyst. [27] This has led us to investigate cross-coupling of aryl chlorides with a range of aryl boronic acids under microwave heating conditions. In this paper we report these results, including our observations on why heavily fluorinated boronic acids are such poor nucleophiles in this chemistry.

## Findings

In our original studies under conventional conditions, it was found that higher and more reproducible yields were obtained using an *in situ* catalyst with a ligand/Pd ratio of > 1.5. Although the active catalyst was proposed to be a mono-ligated Pd(0) species, this can be rationalised in terms of better catalyst stability with more ligand present. The air stable pre-catalyst, **2** was somewhat less effective. In the (faster) reactions described here, good results were also obtained using the pre-catalyst, **2** or using *in-situ* catalysts, with the focus on the use of pre-catalyst **2** due to its greater convenience.

**Scheme 1:**
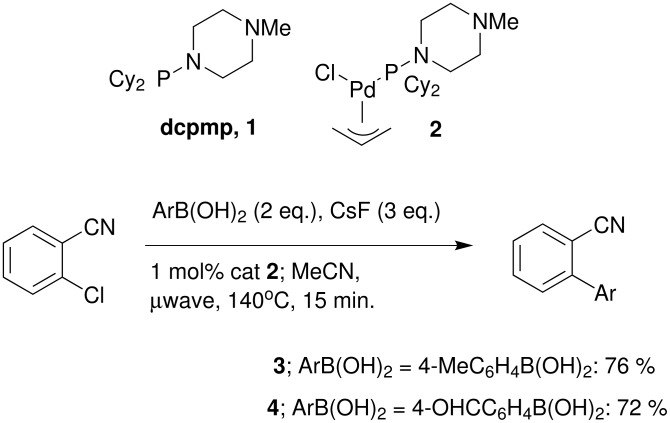
Catalysts used in this study and microwave accelerated cross-coupling of functionalised boronic acids with 2-chlorobenzonitrile.

The cross-coupling of 2-chlorobenzonitrile with both 4-methyl-phenyl boronic acid and 4-formyl boronic acid was investigated, since products **3** and **4** are potential intermediates in the synthesis of the clinically used drugs, Losartan and Valsartan respectively. [28–29] Under conventional heating conditions, attempts to couple 4-formyl benzene boronic acid to 2-chloro-benzonitrile, under conditions that gave high yields using phenyl boronic acid, gave essentially no product (<5% as determined by NMR, GC and TLC analysis). However, reactions carried out in the microwave gave good isolated yields after just 15 minutes at 140°C in acetonitrile. This reason for this difference in reactivity is not clear. However, a number of observations made during our studies suggested that the nature of the boronic acid nucleophile can have a major effect on the productivity of cross-coupling reactions. The majority of studies, including our own, have neglected to fully evaluate this aspect, so we elected to investigate the effect of the boronic acid component using one aryl chloride substrate, 4-chloro-acetophenone. Representative results are compiled in Table 1.

**Table 1:** Cross-coupling of a range of boronic acids with 4-chloroacetophenone.

**Entry^a^**	**ArB(OH)_2_**	**Base**	**Conversion to product (%)**	**Isolated Yield (%)**

1	PhB(OH)_2_	K_3_PO_4_	85	70
2	PhB(OH)_2_	CsF	96	85
3	4-F-C_6_H_4_B(OH)_2_	K_3_PO_4_	88	72
4	4-F-C_6_H_4_B(OH)_2_	CsF ^b^	42	23
5	4-F-C_6_H_4_B(OH)_2_	CsF ^c^	49	N.D.
6	4-F-C_6_H_4_B(OH)_2_	CsF	>98	98
7	3-F-C_6_H_4_B(OH)_2_	K_3_PO_4_	87	73
8	3-F-C_6_H_4_B(OH)_2_	CsF	92	69
9	2-F-C_6_H_4_B(OH)_2_	K_3_PO_4_	82	53
10	2-F-C_6_H_4_B(OH)_2_	CsF	81	64
11	2-MeO-C_6_H_4_B(OH)_2_	CsF	92	80
12	2-MeO-C_6_H_4_B(OH)_2_	CsF^d^	>98	ND
13	4-MeO-C_6_H_4_B(OH)_2_	CsF	82	69
14	3-Napthyl	CsF	64	61
15	2,3,6-F_3_C_6_H_2_B(OH)_2_	CsF	<5	N.D.
16^e^	2,3,6-F_3_C_6_H_2_B(OH)_2_	CsF	<5	N.D.

a: Reactions performed using 1% catalyst **2**, 0.5 mmol of 4-chloro-acetophenone under the conditions described in Scheme 2, unless otherwise stated. Conversion to product refers to the NMR yield of the reactions. Yield refers to isolated yield of pure material after column chromatography, or in the case of entries 3–10, recystallisation. N. D. = not determined. b: DMF used as solvent. c: 1.0 mmol of aryl chloride, 1.5 mmol of boronic acid; 3 mmol of base. d: Reaction time extended to 15 minutes. e: 3-bromoacetophenone used as coupling partner.

**Scheme 2:**
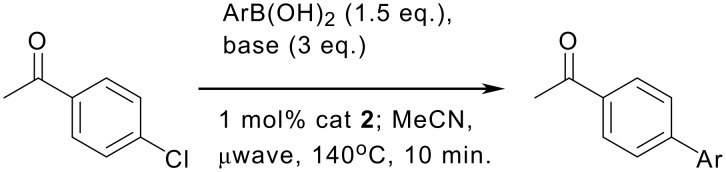
Microwave accelerated cross-coupling of a range of boronic acids with 4-chloroacetophenone.

A range of boronic acids can be coupled with high conversion and good to excellent isolated yield for the biaryl products (see Supporting Information File 1 for experimental data). There has been some interest in the cross-coupling of fluorinated nucleophiles due to the application of fluoroaryl substituents in medicinal chemistry and in liquid crystals. [30–34] The Buchwald group recently reported the first couplings of such substrates with aryl chlorides in high yield. [12] Using our catalyst system, it was pleasing that good yields were also observed with *2*, *3* and *4* substituted fluorophenyl boronic acids but within just 10 minutes of reaction time (Table 1: Entries 6, 8, and 10). Slightly lower yields were encountered with 2-fluorophenyl boronic acid (Entries 9 and 10). An observation that we suspect may be more general is that reactions were less effective if the concentration of boronic acid was too high, probably due to solubility and stirring issues (Compare entries 5 and 6). The preferred procedure used 0.5 mmol of aryl halide and 0.75 mmol of boronic acid in 4–5 ml of solvent. In contrast to the mono-fluorinated phenyl boronic acids, 2,3,6-trifluorophenyl boronic acid failed to give appreciable amounts of product, even with an aryl bromide substrate (Table 1, Entries 15 and 16). Buchwald and co-workers have previously noted the failure of 2,4,6-trifluorophenyl boronic acid to react with aryl chlorides, although the origin of this effect was not identified.

**Scheme 3:**
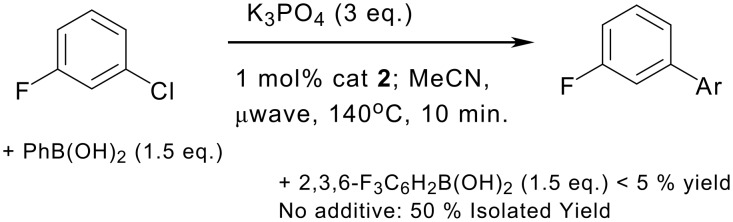
Competition experiment demonstrating the inhibitory effect of 2,3,6-trifluorophenyl boronic acid on Suzuki coupling.

The origin of this low reactivity could be due to slow transmetalation, [35–38] homo-coupling problems,[39] or Pd catalysed protodeboronation,[40] so we have carried out some mechanistic experiments to shed light on this. A competition experiment was set up (Scheme 3): a single vial containing 0.5 mmol of 3-fluoro-chlorobenzene, 0.75 mmol of phenyl boronic acid, 0.75 mmol of 2,3,6 trifluorophenyl boronic acid and 3 equivalents of base was heated under standard conditions and then directly analysed by ^19^F NMR spectroscopy. Neither of the two possible biaryls that could form from the two boronic acids were detected (92% aryl chroride starting material detected). In a separate experiment, under similar conditions, 3-fluoro-chlorobenzene was cross-coupled to phenyl boronic acid, without 2,3,6-trifluorophenyl boronic acid present, albeit in only 50% isolated yield. The presence of the heavily fluorinated boronic acid therefore inhibits the catalyst in some way, as opposed to merely being a weak nucleophile.

**Scheme 4:**
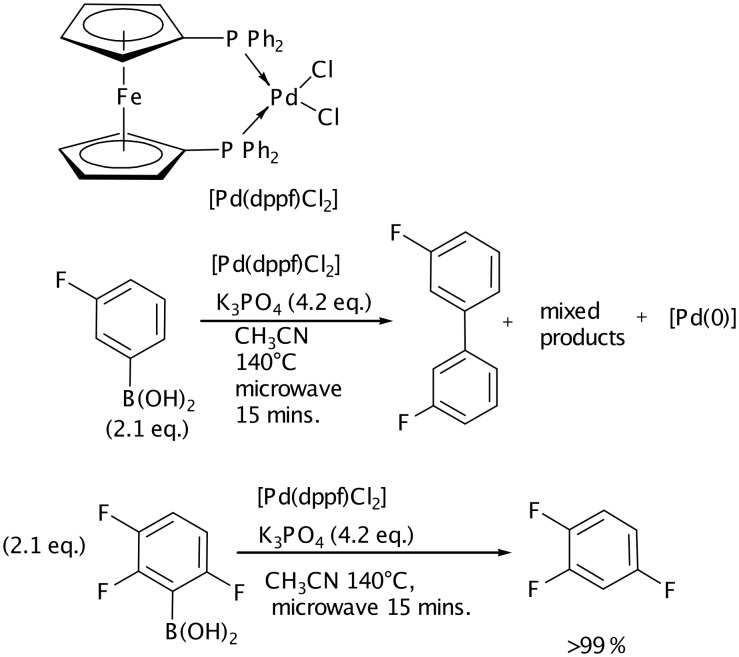
Stoichiometric reactions between fluorinated boronic acids and [Pd(dppf)Cl_2_].

To gain further understanding, stoichiometric experiments between [Pd(dppf)Cl_2_] and boronic acids were carried out (Scheme 4). Given that [Pd(dppf)(Ar)_2_] species rapidly reductively eliminate,[41] successful transmetalation should result in significant amounts of the symmetrical biaryl (alongside a number of other species such as Pd-aryl intermediates, and base-activated aryl boron species). The reaction between 3-fluorophenylboronic acid (2.1 equivalents, 4.2. equivalents of K_3_PO_4_; CD_3_CN, 140°C, 10 min.), and [Pd(dppf)Cl_2_] gives several species when analysed by ^19^F NMR spectroscopy. However, the major product is 3,3'-difluorobiphenyl as established by spiking experiments with a commercial sample.The other species have not been identified, but a further spiking experiment shows that fluorobenzene is not present to any great extent. In contrast, reacting 2,3,6-trifluorophenyl boronic acid with [Pd(dppf)Cl_2_] under the same conditions yields only 1,2,5 trifluorobenzene as the only fluorine containing product. Protodeboronation is therefore the cause of the low reactivity observed here, and is a likely problem in other Suzuki couplings of highly fluorinated substrates. Finally, we stirred 2,3,6-trifluorophenyl boronic acid with K_3_PO_4_ in acetonitrile at room temperature for 15 minutes. The same quantitative formation of the reduced arene was observed even in the absence of Pd catalyst. We therefore propose that Suzuki coupling between this and similar boronic acids is likely to be difficult under any conditions: alternative strategies are required.

In summary, a readily prepared, air stable Pd pre-catalyst derived from the amine-phosphine ligand, **dcpmp** has been found to promote Suzuki coupling between activated aryl chlorides and a range of boronic acids under microwave heating conditions. High yields of the desired biaryls can be obtained in 15 minutes or less. Heavily fluorinated boronic acids do not participate in these Suzuki couplings due to protodeboronation. The accessibility, low cost of the catalyst, short reactions times and convenience of these procedures should make them useful in small scale biaryl synthesis.

## Supporting Information

File 1 Microwave Suzuki biaryl supporting info. Experimental procedures and NMR spectra for the products.
