# Supplementary material for: A convenient catalyst system for microwave accelerated cross-coupling of a range of aryl boronic acids with aryl chlorides
Source: Beilstein J Org Chem. 2007 May 30;3:18. doi: 10.1186/1860-5397-3-18 (PMC1894810; doi:10.1186/1860-5397-3-18)
Supplement: File 1 — Microwave Suzuki biaryl supporting info. Experimental procedures and NMR spectra for the products. [file Beilstein_J_Org_Chem-03-18-s001.pdf]

A convenient catalyst system for microwave accelerated cross-coupling of a range of aryl boronic acids with aryl chlorides.

Matthew L. Clarke,*[a] Marcia B. France,[b] Jose A. Fuentes,[a] Edward J. Milton,[a] Geoffrey J. Roff.[a]

Supporting information

General information**:** all chemicals and solvents are standard laboratory grade, obtained from commercial sources and were used as received. Dry, degassed solvents were used for reactions unless otherwise indicated. Normal grade solvents were used for chromatography. Solvents were removed by rotary evaporation on a Heidolph labrota 4000. Flash column chromatography were performed using Davisil silica gel Fluorochem 60Å, particle size 35-70 micron. All NMR spectra were recorder on Bruker Avance 300 instruments. Mass spectra were recorded on Water Micromass GCT (Time of flight) fitted with lockspray for accurate mass (ESI) or GCT (CI) instruments. All known compounds gave the expected mass spectra. Microwave reactions were carried out in a Biotage® Initiator using 10 ml heavy-walled reactor vials equipped with an air tight seal. The temperature is measured by an infra red temperature probe that measures the temperature on the surface of the vial. The pressure is measured by direct reading of the deflection of the septa on the vial using a load cell behind the inner part of the cavity lid.

The ligand **dcpmp** and precatalyst 2 have been described previously (ref 13&14 in paper). Brief details are reproduced here for convenience. N-methylpiperazine (0.48g; 4.8 mmol) and triethylamine (0.48g; 6.6mmol) were placed in a Schlenk tube equipped with a rubber septum and placed under a dry nitrogen atmosphere. Dry diethyl ether (20ml) was then added. The solution was cooled down to 0 °C and dicyclohexylchlorophosphine (1g; 4.3mmol) was added dropwise. The solution was then stirred at room temperature overnight, after which time phosphorus NMR demonstrated a quantitative conversion to the desired ligand ($^{31}P\{^1H\}$-NMR (121.5MHz, C_6D_6) δ 76.12). If required the ligand can be isolated by Schenk filtration and removal of volatiles and stored for long periods of time (many months) under a nitrogen atmosphere. To prepare air stable precatalyst, 8, 10ml of the ligand solution were transferred to a Schlenk flask containing allylpalladium chloride dimer (0.2g; 0.0014mol) and the resulting solution stirred for 3 hours. The yellow precipitate that formed was filtered and washed with ether. ($^{31}P\{^1H\}$-NMR (121.5MHz; $CDCl_3$) δ 96.87).

Example Suzuki procedure: Synthesis of 4-acetyl-2'-methoxybiphenyl: To a clean dry microwave tube containing catalyst **2** (1 mol %) was added 2-methoxyphenylboronic acid (114mg, 0.75mmol) and cesium fluoride (228mg, 1.5mmol). The tube was sealed and the air displaced with nitrogen before the addition of dry degassed acetonitrile (2ml) followed by 4-chloroacetophenone (65μl, 0.5mmol). The mixture was heated in a microwave at 140°C for 10 minutes. ^1H NMR of the products described here proved to be a useful method to estimate conversion, since the acetyl protons of starting material, product, and acetophenone all have different chemical shifts. In this case a 92% conversion to the desired product was observed. The mixture was concentrated *in vacuo* and flash column chromatography (eluent: hexane/ethyl acetate 5/1) gave 90mg (80%) of a white solid. Other biaryls were prepared and isolated in a similar manner. The compounds produced are known, and were identified by comparison of NMR and mass spectral data with the literature.

Selected NMR data:

1-(2'-methoxybiphenyl-4-yl)ethanone

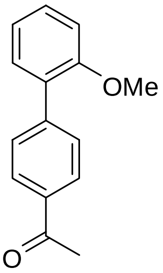

^{1}H NMR (300MHz, $CDCl_3$) δ = 2.64 (3H, s), 3.83 (3H, s), 7.00-7.08 (2H, m), 7.32-7.38 (2H, m), 7.64 (2H, d *J* = 8.4 Hz), 8.00 (2H, d *J* = 8.4 Hz); ^{13}C NMR (75MHz, $CDCl_3$) δ = 26.7, 55.6, 111.4, 121.0, 129.5, 130.8, 128.1, 129.8, 135.5, 143.6, 156.5, 197.9;

V. Farina, B. Krishnan, D.R. Marshall, G.P. Roth, *J. Org. Chem.*, **1993**, *58*, 5434.

4'-Methyl-biphenyl-2-carbonitrile

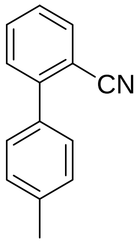

^{1}H NMR (360 MHz, $CDCl_3$) δ = 2.43 (3H, s), 7.30-7.66 (7H, m), 7.76 (1H, dd *J* = 1.38, 7.72); ^{13}C NMR (75 MHz, $CDCl_3$) δ = 21.7, 111.6, 119.3, 127.7, 129.0, 129.9, 130.4, 133.2, 134.1, 135.7, 139.1, 145.9.

Q. Dai, W. Gao, D. Liu, L. M. Kapes, X. Zhang. *J. Org. Chem.* **2006**, *71*, 3928.

4'-Formyl-biphenyl-2-carbonitrile

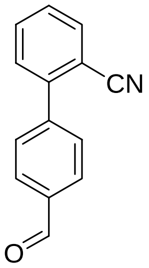

^{1}H NMR (360 MHz, $CDCl_3$) δ = 7.42-7.49 (2H, m), 7.60-7.68 (3H, m), 7.72-7.75 (1H, m), 7.92-7.96 (2H, m), 10.02 (1H, s); ^{13}C NMR (75 MHz, $CDCl_3$) δ = 111.7, 118.7, 129.0, 130.0, 130.4, 130.5, 133.5, 134.4, 136.6, 144.3, 144.4, 192.1.

J. Kristensen, M. Lysén, P. Vedsø, M. Begtrup. *Org. Lett.* **2001**, *3*, 1435.

1-(4'-methoxybiphenyl-4-yl)ethanone

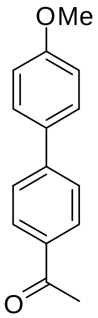

^{1}H NMR (300MHz, $CDCl_3$) δ = 2.64 (3H, s), 3.88 (3H, s), 7.00 (2H, d *J* = 9.0 Hz), 7.59 (2H, d *J* = 9.0 Hz), 7.63 (2H, d *J* = 8.7 Hz), 8.02 (2H, d *J* = 8.7 Hz); ^{13}C NMR (75MHz, $CDCl_3$) δ = 26.7, 55.4, 111.4, 126.6, 128.4, 129.0, 132.3, 135.3, 145.4, 160.0, 197.7;

V. Farina, B. Krishnan, D.R. Marshall, G.P. Roth, *J. Org. Chem.*, **1993**, *58*, 5434.

1-(4-(naphthalen-2-yl)phenyl)ethanone

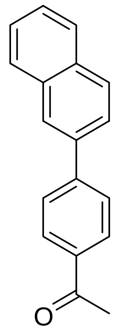

^{1}H NMR (300MHz, $CDCl_3$) δ = 2.68 (3H, s), 7.52-7.58 (2H, m), 7.76-8.10 (5H, m), 7.83 (2H, d *J* = 8.5 Hz), 8.09 (2H, d *J* = 8.5 Hz); ^{13}C NMR (75MHz, $CDCl_3$) δ = 26.7, 127.5, 129.0, 125.2, 126.4, 126.5, 126.6, 127.7, 128.4, 128.8, 133.1, 133.6, 135.9, 137.2, 145.7, 198.7;
J-P Duan, C-H Cheng, *Organometallics*, **1995**, *14*, 1608.

1-(biphenyl-4-yl)ethanone

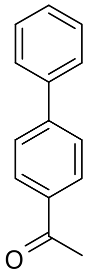

^{1}H NMR (300MHz, $CDCl_3$) δ = 2.66 (3H, s), 7.41-7.69 (4H, m), 7.71 (2H, d *J* = 8.7 Hz), 8.05 (2H, d *J* = 8.7 Hz); ^{13}C NMR (75MHz, $CDCl_3$) δ = 26.7, 127.2, 127.3, 128.3, 129.0, 135.9, 145.8, 197.8
E.M. Schulman, K.A. Christensen, D.M. Grant, C. Walling, *J. Org. Chem.*, **1974**, *39*, 2686.

1-(2'-fluorobiphenyl-4-yl)ethanone

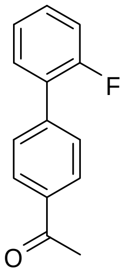

^{1}H NMR (300MHz, $CDCl_3$) δ =2.66 (3H, s), 7.16-7.50 (4H, m), 7.67 (2H, dq *J* = 1.8 Hz, 8.7 Hz), 8.05 (2H, dt *J* = 1.8 Hz, 8.7 Hz); ^{13}C NMR (75MHz, $CDCl_3$) δ = 27.1, 116.8 (d, *J* = 22.7 Hz), 125.0 (d, *J* = 3.9 Hz), 128.9, 129.6 (d, *J* = 3.3 Hz), 130.3 (d *J* = 8.3 Hz), 131.0 (d *J* = 3.3 Hz), 136.5, 141.0, 160.0 (d, *J* = 248.8 Hz), 198.1 ; ^{19}F {^{1}H} NMR (282 MHz, $CDCl_3$) δ= -117.93
W. Solodenko, K. Mennecke, C. Vogt, S. Gruhl, A. Kirshning; *Synthesis*, **2006**, *11*, 1873.

1-(3'-fluorobiphenyl-4-yl)ethanone

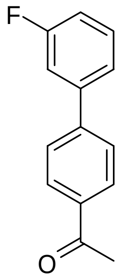

^{1}H NMR (300MHz, $CDCl_3$) δ = 2.66 (3H, s), 7.27-7.49 (4H, m), 7.68 (2H, d *J* = 8.7 Hz), 8.05 (2H, d *J* 8.7 Hz); ^{13}C NMR (75MHz, $CDCl_3$) δ = 26.7, 114.2 (d, *J* = 22.1 Hz), 115.1 (d, *J* = 21.0 Hz), 125.9 (d, *J* = 3.3 Hz), 127.3, 129.0, 130.5 (d, *J* = 8.3 Hz), 136.4, 142.1 (d, *J* = 7.7 Hz), 144.0, 163.2 (d, *J* = 246.0 Hz), 198.1 ;^{19}F {^{1}H} NMR (282 MHz, $CDCl_3$) δ = -113.02
N.J. Thompson, J.W. Goodby, K.J. Toyne, *Mol. Cryst. Liq. Sci. Technol. Sect. A.*, **1992**, *214*, 81.

1-(4'-fluorobiphenyl-4-yl)ethanone

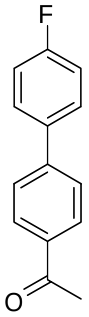

^{1}H NMR (300MHz, $CDCl_3$) δ = 2.65 (3H, s), 7.58-7.64 (4H, m), 7.66 (2H, d *J* = 8.7 Hz), 8.04 (2H, d *J* = 8.7 Hz); ^{13}C NMR (75MHz, $CDCl_3$) δ = 26.7, 115.9 (d, *J* = 21.6 Hz), 127.1, 128.9, 129.0, 135.9, 136.0 (d, *J* = 3.3 Hz), 144.7, 163.0 (d, *J* = 248.2 Hz), 198.1 ;^{19}F {^{1}H} NMR (282 MHz, $CDCl_3$) δ= -114.47
J-H Li, W-J Liu, *Org. Lett.*, **2004**, *6*, 2809.

3-fluorobiphenyl

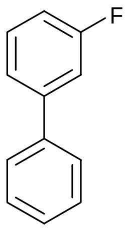

^{1}H NMR (300MHz, $CDCl_3$) δ = 6.90-6.97 (1H, m), 7.16-7.22 (1H, m), 7.24-7.38 (5H, m), 7.44-7.50 (2H, m); ^{13}C NMR (75MHz, $CDCl_3$) δ = 114.31 (d, *J* = 1.7 Hz), 114.61, 123.19 (d, *J* = 2.8 Hz), 127.54, 128.28, 129.32, 130.65 (d, *J* = 8.4 Hz), 140.35, 143.93 (d, *J* = 7.6 Hz), 162.0, 165.26; ^{19}F NMR {^{1}H} (282 MHz, $CDCl_3$) δ = -113.54.
E. Anklam, K.D. Asmus, L.W. Robertson, *J. Fluorine Chem.*, **1988**, *39*, 209.

3,3'-difluorobiphenyl

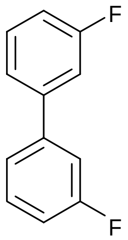

^{1}H NMR (300MHz, $CDCl_3$) δ = 6.90-7.18 (1H, m), 7.12-7.51 (5H, m), 7.72-7.82 (1H, m), 7.84-7.98 (1H, m); ^{13}C NMR (75MHz, $CDCl_3$) δ = 114.47 (d, *J* = 21.1 Hz), 115.05 (d, *J* = 21.1 Hz), 123.17 (d, *J* = 2.8 Hz), 130.84 (d, *J* = 8.4 Hz), 142.60 (dd, *J* = 2.2, 7.7 Hz), 162.03, 165.29; ^{19}F {^{1}H} NMR (282 MHz, $CDCl_3$) δ = -113.32.
M.A. Cooper, H.E. Weber, S.L. Manatt, *J. Am. Chem. Soc.*, **1971**, *93*, 2369.

1,3,4-trifluorobenzene

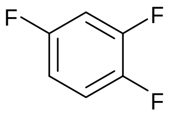

^{1}H NMR (300MHz, $CDCl_3$) δ = 6.90-6.96 (1H, m), 7.01-7.19 (1H, m), 7.20-7.30 (1H, m); ^{19}F {^{1}H} NMR (282 MHz, $CDCl_3$) δ = -117.10 (dd, *J* = 3 Hz, 15 Hz), -135.71 (dd, *J* = 3 Hz, 15 Hz), -145.33 (dd, *J* = 3 Hz, 15 Hz).
P.L Coe, A.L Stuart, D.J Moody, *J. Chem. Soc., Perkin Trans. 1*, **1998**, 1807.
